# Large Language Model–Enhanced Drug Repositioning Knowledge Extraction via Long Chain-of-Thought: Development and Evaluation Study

**DOI:** 10.2196/77837

**Published:** 2025-10-07

**Authors:** Hongyu Kang, Jiao Li, Li Hou, Xiaowei Xu, Si Zheng, Qin Li

**Affiliations:** 1 School of Medical Technology, Beijing Institute of Technology, No. 5 Zhongguancun South Street, Haidian District, Beijing, 100081, China, 86 13693067129; 2 Medical Information Innovation Research Center, Institute of Medical Information, Chinese Academy of Medical Sciences and Peking Union Medical College, Beijing, China

**Keywords:** drug repositioning, knowledge extraction, large language models, long chain-of-thought, reinforcement learning

## Abstract

**Background:**

Drug repositioning is a pivotal strategy in pharmaceutical research, offering accelerated and cost-effective therapeutic discovery. However, biomedical information relevant to drug repositioning is often complex, dispersed, and underutilized due to limitations in traditional extraction methods, such as reliance on annotated data and poor generalizability. Large language models (LLMs) show promise but face challenges such as hallucinations and interpretability issues.

**Objective:**

This study proposed long chain-of-thought for drug repositioning knowledge extraction (LCoDR-KE), a lightweight and domain-specific framework to enhance LLMs’ accuracy and adaptability in extracting structured biomedical knowledge for drug repositioning.

**Methods:**

A domain-specific schema defined 11 entities (eg, drug, disease) and 18 relationships (eg, treats, is biomarker of). Following the established schema architecture, we constructed automatic annotation based on 10,000 PubMed abstracts via chain-of-thought prompt engineering. A total of 1000 expert-validated abstracts were curated into a drug repositioning corpus, a high-quality specialized corpus, while the remaining entries were allocated for model training purposes. Then, the proposed LCoDR-KE framework combined supervised fine-tuning of the Qwen2.5-7B-Instruct model with reinforcement learning and dual-reward mechanisms. Performance was evaluated against state-of-the-art models (eg, conditional random fields, Bidirectional Encoder Representations From Transformers, BioBERT, Qwen2.5, DeepSeek-R1, OpenBioLLM-70B, and model variants) using precision, recall, and *F*_1_-score. In addition, the convergence of the training method was assessed by analyzing performance progression across iteration steps.

**Results:**

LCoDR-KE achieved an entity *F*_1_ of 81.46% (eg, drug 95.83%, disease 90.52%) and triplet *F*_1_ of 69.04%, outperforming traditional models and rivaling larger LLMs (DeepSeek-R1: entity *F*_1_=84.64%, triplet *F*_1_=69.02%). Ablation studies confirmed the contributions of supervised fine-tuning (8.61% and 20.70% *F*_1_ drop if removed) and reinforcement learning (6.09% and 14.09% *F*_1_ drop if removed). The training process demonstrated stable convergence, validated through iterative performance monitoring. Qualitative analysis of the model’s chain-of-thought outputs showed that LCoDR-KE performed structured and schema-aware reasoning by validating entity types, rejecting incompatible relations, enforcing constraints, and generating compliant JSON. Error analysis revealed 4 main types of mistakes and challenges for further improvement.

**Conclusions:**

LCoDR-KE enhances LLMs’ domain-specific adaptability for drug repositioning by offering an open-source drug repositioning corpus and a long chain-of-thought framework based on a lightweight LLM model. This framework supports drug discovery and knowledge reasoning while providing scalable, interpretable solutions applicable to broader biomedical knowledge extraction tasks.

## Introduction

Drug repositioning, also known as drug repurposing, has emerged as a pivotal strategy in pharmaceutical research, enabling the discovery of novel therapies for existing drugs [1]. This approach significantly facilitates the drug development process, shortens the required time, and reduces the cost [2]. However, biomedical information relevant to drug repositioning is often complex and dispersed across various sources [3], such as literature, clinical trials, and databases. These valuable resources remain underutilized because of the challenges associated with knowledge extraction [4,5]. Extracting knowledge from biomedical literature, such as entities, relationships, and structured triplets, can facilitate drug repositioning research by uncovering hidden connections between drugs and diseases [6]. Recent studies have demonstrated the effectiveness of knowledge extraction in supporting knowledge discovery in drug repositioning. For example, Bang et al [7] developed a semantic multilayer guilt-by-association approach to extract drug-disease associations from large-scale biomedical corpora, while Huang et al [8] leveraged TxGNN, a graph foundation model for zero-shot drug repurposing, to identify therapeutic candidates even for diseases with limited treatment options or no existing drugs. These advancements underscore the importance of automated knowledge extraction in identifying potential drug candidates for the treatment of complex, multifactorial diseases.

Traditional knowledge extraction methods have evolved from manual annotation and rule-based techniques to machine learning and deep learning approaches. Early methodologies relied heavily on expert-curated databases and handcrafted rules, which lacked scalability while being precise [9,10]. Machine learning models, such as conditional random fields (CRF), introduced statistical learning to automate entity recognition and relationship extraction [11-13]. More recently, deep learning models, including BERT and transformer-based architecture, have achieved remarkable performance in biomedical natural language processing tasks. For example, domain-specific language models, such as BioBERT [14], PubMedBERT (trained on biomedical literature) [15], ClinicalBERT (trained on the MIMIC-III dataset) [16], and PharmBERT (trained on prescription drug labeling) [17], have been further developed. Despite their success, these methods still have several limitations. They often require extensive labeled datasets for training, which are costly and labor-intensive to obtain. Furthermore, deep learning models demonstrate limited generalizability across diverse biomedical domains [18] and often overlook implicit knowledge that extends beyond surface-level textual patterns [19]. As a result, refining extraction methodologies to achieve greater adaptability and performance continues to be an important focus in drug repositioning studies.

Large language models (LLMs) have emerged as a promising solution for biomedical knowledge extraction, demonstrating superior performance across various natural language processing tasks. Models such as ChatGPT (OpenAI) [20], LlaMA (Meta) [21], GLM (Tsinghua) [22], Qwen (AlibabaCloud) [23], and Deepseek [24] have been extensively used in biomedical text mining because of their advanced semantic comprehension and text generation capacities. By leveraging key methodologies, including pretraining [25], prompt engineering [26], and domain-specific fine-tuning [27], these models demonstrate improved accuracy and adaptability for specialized tasks [28], such as named entity recognition [29] and association prediction [30]. Xie et al [31] developed Me-LLaMA through continual pretraining and instruction tuning of LLaMA2 models and outperformed existing open medical LLMs on 6 text analysis tasks. Hao et al [26] developed and evaluated MedScaleNER, a task-oriented prompt framework, advancing the application of LLMs and prompt engineering for specialized named entity recognition tasks in Chinese medical literature. Recent advances such as BioGPT [32] and OpenBioLLM-70B [33] have demonstrated the potential of large-scale generative pretrained transformers in enhancing biomedical text generation and domain-specific knowledge mining. Focusing on a more specific biomedical task, Yuan et al [34] introduced BioFocal-DDI, a framework combining BioGPT for data augmentation, BioBERT, and BiLSTM for contextual and sequential feature extraction to optimize drug-drug interaction extraction.

Compared to traditional methods, LLMs demonstrate superior knowledge extraction capabilities, including better generalization, reduced reliance on annotated data, and stronger contextual comprehension. Nevertheless, persistent challenges [35,36], including model hallucination, output accuracy, interpretability, and high computational demands, must be addressed. For drug repositioning applications, these issues can be mitigated through gold-standard corpus validation, careful prompt engineering, and chain-of-thought (CoT) [37] methodologies. Chen et al [38] leveraged retrieval‐augmented generation combined with instruction prompting to build LLMs specialized for osteoarthritis diagnosis and treatment Q&A, providing reliable clinical decision support to health care professionals.

While LLMs demonstrate promise in biomedical knowledge extraction, challenges persist in domain-specific adaptability, particularly in drug repositioning, where sparse annotated data, semantic ambiguity, and cross-sentence dependencies hinder accurate knowledge inference [39,40]. To address these limitations, we proposed long chain-of-thought (LCoT) for drug repositioning knowledge extraction (LCoDR-KE), a lightweight framework that combined LCoT prompting, supervised fine-tuning (SFT), and reinforcement learning (RL). To enhance the performance of knowledge extraction, our approach also introduced a dual-level reward mechanism to guide knowledge extraction and a high-quality drug repositioning corpus (DrugReC), meticulously curated from biomedical publications in PubMed. Through comparative and ablation studies, we validated the framework’s superiority over traditional models and its competitive performance against state-of-the-art LLMs, offering a scalable, interpretable solution for accelerating drug discovery and biomedical knowledge inference.

## Methods

### Overview

**Figure 1.**
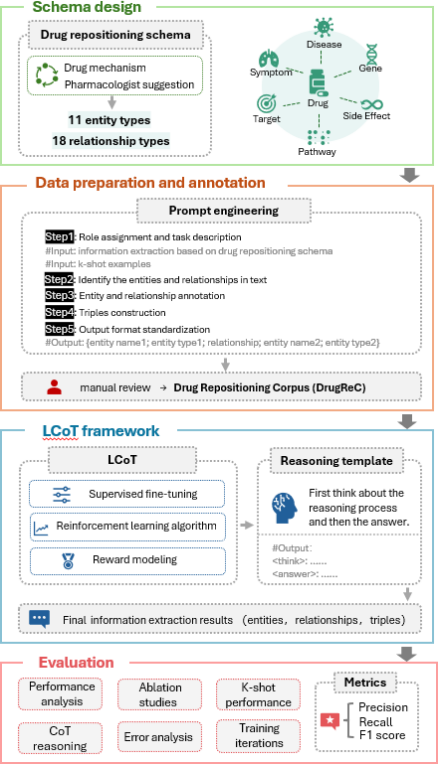
Workflow of long chain-of-thought for drug repositioning knowledge extraction (LCoDR-KE). CoT: chain-of-thought; LCoT: long chain-of-thought.

The workflow of the proposed *LCoDR-KE* is illustrated in Figure 1 and consisted of 4 main stages: drug repositioning schema design, data preparation and gold standard annotation, implementation of LCoT framework, and in-depth evaluation.

- *Drug repositioning schema design*: This stage established a structured schema for drug repositioning, defining 11 entity types (eg, drug, gene, disease, target) and 18 relationship types. The schema provided a standardized ontology that enabled accurate knowledge extraction while maintaining conceptual consistency across the domain.

- *Data preparation and annotation*: We first annotated a scale of 10,000 PubMed abstracts via CoT prompt engineering, incorporating role definitions and k-shot demonstrations. Among these, 1000 abstracts were subjected to meticulous manual annotations, resulting in a high-quality corpus specifically curated for the task of drug repurposing, DrugReC. The remaining 9000 abstracts were used as part of the training data in this study.
- *Implementation of the LCoT framework:* We first conducted SFT to establish a strong output with explicit reasoning chains for LLM. Following this, we applied Group Relative Policy Optimization (GRPO), an RL strategy guided by reward modeling. Tailored reward functions were specifically designed for drug repositioning entity and triple recognition, enabling more accurate and structured output aligned with domain-specific requirements.
- *Evaluation and performance analysis*: We evaluated the model’s effectiveness through performance comparisons, ablation studies, k-shot performance, reward parameter optimization, CoT reasoning patterns, steps of training iterations, and error analysis.

### Drug Repositioning Schema Design

We developed a multidimensional knowledge model by conducting in-depth analyses of drug mechanisms [41-43] while precisely defining core entity dimensions and their relationships. We conducted comprehensive analyses of drug labels, literature, and multiple biomedical databases (including DrugBank, CTD, PharmGKB, and DisGeNET) to establish foundational knowledge. In addition, this multidimensional knowledge framework was subsequently refined and validated through expert consultation, incorporating specialized domain knowledge from pharmacology, bioinformatics, clinical medicine, and computational biology.

### Data Preparation

A total of 10,000 PubMed abstracts were extracted using a 4-step filtering strategy to ensure quality and domain relevance:

1. Initial search: Articles published between January 1, 2015, and January 1, 2025, were retrieved via PubMed using Medical Subject Headings (MeSH) terms and keywords related to drug repositioning, drug mechanisms, and pharmacology.
2. Journal filtering: We retained articles published in top-tier pharmacological journals ranked in Q1 of the Journal Citation Reports (Multimedia Appendix 1).
3. Relevance filtering: Abstracts were kept if the title or abstract mentioned at least one drug or disease entity from a standard drug repositioning dataset [44].
4. Length filtering: Abstracts under 50 characters were excluded to ensure basic content adequacy.

This rigorous selection process (detailed in a PRISMA [Preferred Reporting Items for Systematic Reviews and Meta-Analyses] flow diagram in Multimedia Appendix 1) ensured the inclusion of the latest high-quality research articles relevant to pharmacological mechanism and repurposing strategies.

### Data Annotation With CoT-Prompt Engineering

Owing to the lack of annotated datasets for drug repositioning knowledge extraction, we constructed an annotated corpus from 1000 PubMed abstracts mentioned earlier. The prompt was evaluated on DeepSeek-R1 [45] via CoT [46], which required LLMs to generate coherent intermediate reasoning steps leading to a final answer [47].

We summarized the execution steps and proposed a task-specific prompt as follows and outlined them in Multimedia Appendix 2.

- Step 1: Role assignment and task description. Initialized the LLM as a biomedical annotation specialist, explicitly defining the drug repositioning schema and incorporating k-shot demonstrations to establish contextual understanding of target entities and semantic relationships.
- Step 2: Identification of entity and relationship types. Identified candidate entity types and contextual relationship types in text according to predefined schema.
- Step 3: Entity and relationship annotation. Based on the categories identified in step 2, this step extracted specific entities (eg, “Metformin” as *drug, “Diabetes” as disease*) and their contextual relationships (eg, *treats*) from text.
-  Entity annotation principles. (1) Nonoverlapping: A single string cannot be assigned to multiple entity types. (2) Nonnesting: An entity should not contain another entity within its span. (3) Minimal punctuation and conjunction: Entities should exclude unnecessary punctuation or connecting words.
-  Relationship annotation principles. (1) Intrasentence priority: Triplets within a single sentence are preferred; cross-sentence triplets are permitted only if no valid intrasentence relation exists. (2) Unidirectional relations: Only 1 directional relation is retained between any 2 related entities.
- Step 4: Triplets construction. Extracted drug repositioning entities and their contextual associations into semantically coherent triplets.
- Step 5: Output format standardization. Specified the output format and standards, setting clear expectations for the output as {Entity1; Type1; Relationship; Entity2; Type2} in JSON format.

To ensure the quality of the DrugReC, we manually reviewed the standard results from 1000 abstracts as well. A total of 3 annotators in pharmacology, clinical medicine, and bioinformatics were recruited, with 2 conducting cross-review and the third senior expert adjudicating conflicting or ambiguous cases. The corpus was subsequently partitioned into a training set (n=800) for RL algorithm development and a test set (n=200) to evaluate model generalizability. The remaining 9000 abstracts were used as training data for SFT.

### Long Chain-of-Thought

#### LLM Deployment

The Qwen2.5-7B-Instruct model [48], developed by Alibaba Cloud, represented a significant advancement in the Qwen series of LLMs, specifically optimized for semantic comprehension and complex task processing. This 7-billion-parameter transformer-based architecture demonstrated exceptional performance in specialized applications, including knowledge extraction [49] and conversational interaction [50] scenarios. Meanwhile, an extended context window of 128,000 tokens facilitated precise capture of fine-grained semantic features within textual data.

For the Qwen2.5-7B-Instruct setup, we used temperature sampling with the parameter of 0.02. A lower temperature parameter can limit the model to make excessive creative jumps, thus ensuring accurate and consistent outputs. Meanwhile, we set the max_token parameter to 16,384 while leaving all other hyperparameters at their default values. The used hyperparameters during model training included a training batch size of 8, a learning rate of 1e−6, a cutoff length of 16,384, and a gradient accumulation step of 1. The model was trained on the Wuwen Xinqiong platform. Training the 7B model with 8× NVIDIA A100 40GB GPUs took approximately 40 hours, with a total cost of approximately 1000 RMB (US $140.47).

#### Cold-Start SFT

To address the output incoherence and unstable reasoning patterns caused by directly initializing RL [51] in foundation LLMs, we implemented cold-start SFT [52] with CoT data from 9000 PubMed literature as mentioned earlier. These domain-specific annotated triplets were used to optimize model parameters under a supervised learning framework, enabling the model to progressively acquire human-aligned reasoning patterns. This phase implemented multistep reasoning paths and strict output formats, significantly enhancing the model’s complex problem decomposition capacity and intermediate step interpretability. In brief, the approach of cold-start SFT provided stable initialization for subsequent RL stages.

#### RL Algorithm

To reduce the training costs associated with RL, we used GRPO to further optimize reasoning paths through self-iteration in an unannotated environment [53]. Specifically, GRPO generated multiple candidate outputs $\left\{o_{1},o_{2},\ldots o_{G}\right\}$ from the old policy $\pi_{\theta_{old}}$ and then optimized the policy model $\pi_{\theta }$ by maximizing the following objective:


(1) $$\zeta_{GPRO}(\theta )=E\left[\sum_{i=1}^{G}\left(\min\left(\frac{\pi_{\theta }(o_{i})}{\pi_{\theta_{\text{old}}}(o_{i})}A_{i},\;clip\left(\frac{\pi_{\theta }(o_{i})}{\pi_{\theta_{\text{old}}}(o_{i})},\;1-\varepsilon ,\;1+\varepsilon \right)A_{i}\right)\right)-\beta D_{KL}\;\left(\pi_{\theta }\;\|\;\pi_{\text{ref}}\right)\right]$$

(2) $$D_{KL}\left(\pi_{\theta }\|\pi_{ref}\right)=\frac{\pi_{\theta_{ref}}\left(o_{i}|q\right)}{\pi_{\theta }\left(o_{i}|q\right)}-log\frac{\pi_{\theta_{ref}}\left(o_{i}|q\right)}{\pi_{\theta }\left(o_{i}|q\right)}-1$$

and the advantage $A_{i}$ was defined as follows:


(3) $$A_{i}=(r_{i}-\mu_{group})/\sigma_{group}$$

where $r_{i}$ was the raw reward for the *i*th answer and $\mu_{group}$ and $\sigma_{group}$ represented the mean and SD, respectively, of rewards within the candidate outputs. The group-wise contrastive mechanism mitigated absolute reward scale bias while encouraging high-quality reasoning.

For the task of drug repositioning knowledge extraction, this optimization process facilitated complex cognitive mechanisms based on a high-performance RL training framework, EasyR1 [54], leading to enhancement of task-specific reasoning performance.

#### Reward Modeling

Subsequently, we proposed a novel dual-reward mechanism specifically for drug repositioning knowledge extraction to guide model self-evolution. This mechanism integrates 2 complementary reward types: (1) an accuracy reward targeting factual correctness in entity and triplet extraction and (2) a structural format reward focusing on the integrity and interpretability of the model’s reasoning process through structured tag compliance.

### Accuracy Reward Mechanism

This mechanism established a multidimensional evaluation that compares model outputs with annotated references. Dynamic scoring rules for entity recognition and triplet extraction are presented in Table 1.

According to these scoring rules, the accuracy reward was as follows:


(4) $$R_{acc}=\alpha S_{entity}+\beta S_{triplet}+\gamma S_{rare}$$

where $S_{entity}$ was accuracy reward of entity recognition, $S_{triplet}$was accuracy reward of triplet extraction, and $S_{rare}$ was reward of rare entity or relationship. This hierarchical award mechanism effectively balanced comprehensiveness and granularity in drug repositioning knowledge extraction.

**Table 1.** Scoring rules of accuracy reward.

Reward factor and detail	Reward mechanism
Entity recognition	
Exact match: boundary and type fully consistent	Score rewarded (+)
Partial overlap: correct type with boundary overlap	Partial rewarded (+)
False positive: misrecognition and false negative: omission	No score (0)
Triplet extraction	
Correct triplet	Score rewarded (+)
Dependency error: incorrect entity in relation‌	No score (0)
Others	
Rare entity/relationship	Score rewarded (+)

### Structural Format Reward

To enhance the model’s step-by-step reasoning, we also introduced an XML tag–guided mechanism (Table 2) that enforced structured output. It was required to encapsulate complete reasoning processes within “<think>……</think>” tags and nested <step> tags, enabling multilevel logical decomposition.

According to these scoring rules, the structural format reward was as follows:


(5) $$R_{fmt}=\delta S_{tag}+\varepsilon S_{length}$$

where $S_{tag}$ was format reward of tag integrity and $S_{length}$ was format reward of thinking length. This approach ensured machine-parsable outputs while enhancing the interpretability of inference pathways.

**Table 2.** Scoring rules of structural format reward.

Reward factor and detail	Reward mechanism
Tag integrity‌	
Missing/redundant tags‌	No score (0)
Tag closure disorder‌: closing tag appears before opening tag	No score (0)
Thinking length	
Underlength/overlength content	No score (0)

### In-Depth Evaluation and Comparison

To comprehensively assess the performance of LCoDR-KE, we conducted an in-depth analysis.

First, comparative experiments were conducted to benchmark LCoDR-KE against traditional machine learning models (eg, CRF), general-purpose pretrained models (BERT), domain-specific biomedical models (BioBERT), and state-of-the-art LLMs, including ChatGPT-4, Llama3, QIANWEN, and variants with different parameter scales. These comparative analyses served to validate the effectiveness of our proposed model against established benchmarks, thereby highlighting its superior performance in addressing specialized biomedical knowledge extraction problems.

Second, ablative experiments were designed to evaluate the individual contributions of the key technological advancements, including high-quality data-driven prompt engineering and LCoT reasoning mechanism. By removing or substituting these modules, the study quantified their independent contributions and synergistic effects on task performance.

Finally, we conducted an error analysis by manually reviewing 100 randomly selected abstracts from the model outputs to identify common types of mistakes and areas for future algorithmic improvements.

Meanwhile, we used precision, recall, and *F*_1_-score for model evaluations. Precision quantifies the proportion of correct identifications among all model predictions, reflecting the reliability of positive results. Recall evaluates the coverage for true positive instances, indicating the comprehensiveness of knowledge extraction. The *F*_1_-score, as the harmonic mean of precision and recall, provides a balanced assessment that mitigates the impact of omissions and false extraction, thereby offering a comprehensive evaluation. For rigorous validation, we compared model outputs against manually annotated corpus using exact string matching, which ensured high quality of model performance.

### Ethical Considerations

This study did not involve human participants or animal experiments. All data used in the research, including biomedical literature and structured databases, were publicly available and deidentified before analysis. Therefore, ethics approval was not required in accordance with institutional and international research ethics policies.

## Results

### Knowledge Representation for Drug Repositioning

Drug repositioning schema (summarized in Multimedia Appendix 3) encompassed 11 entity categories, including core entities (drug, disease, and target) along with extended dimensions, such as side effect, gene, biomarker, symptom, complication, anatomical structure, clinical examination, and treatment. Furthermore, 18 relationship categories were defined in detail to comprehensively characterize semantic features among multilevel entities, such as “treat...,” “is target of,” “is side_effect of,” “is biomarker of.” This framework substantially enhanced semantic representation capabilities and facilitated the discovery of latent associations in drug repositioning research.

### Statistics of DrugReC

Via defined prompt engineering and manual review, we constructed a high-quality DrugReC, specially for drug repurposing text mining. This corpus, annotated from 1000 PubMed abstracts, contained 11 entity types and 18 relationship types closely related to drug repositioning. After the concordance test and revision, a total of 9329 entities and 4879 triplets consisted of this standard dataset (presented in Table 3). Among the entity types, *disease* (19.10%) and *drug* (15.80%) were the most frequently represented, followed by *anatomy* (13.62%), *test* (10.32%), and *biomarker* (10.26%). Less prevalent but critical categories included *complication*, *target*, *treatment*, *symptom*, *gene*, and *side effect*. Regarding knowledge triplets, *is_examination_for* (18.16%), *treat* (17.20%), and *is_biomarker_of* (17.16%) together accounted for more than half of all triplets, emphasizing the corpus’s focus on clinically relevant associations. This distribution reflected DrugReC’s design to support comprehensive text mining efforts in drug repositioning by capturing key therapeutic and diagnostic linkages. We have made this corpus publicly available on GitHub with Apache License version 2.0, providing a high-quality data foundation to advance biomedical informatics mining.

Then, this dataset was randomly divided at the document level into training (80%) and test (20%) subsets, with detailed statistical characteristics of both partitions provided in Tables3  4.

**Table 3.** Statistics of entities in training and test set.

Entity type	Total, n (%)	Training set, n (%)	Test set, n (%)
disease	1782 (19.10)	1426 (19.11)	356 (19.08)
drug	1474 (15.80)	1179 (15.80)	295 (15.81)
anatomy	1271 (13.62)	1017 (13.63)	254 (13.61)
test	963 (10.32)	770 (10.32)	193 (10.34)
biomarker	957 (10.26)	766 (10.26)	191 (10.24)
complication	644 (6.90)	515 (6.90)	129 (6.91)
target	583 (6.25)	466 (6.24)	117 (6.27)
treatment	561 (6.01)	449 (6.02)	112 (6.00)
symptom	490 (5.25)	392 (5.25)	98 (5.25)
gene	362 (3.88)	290 (3.89)	72 (3.86)
side effect	242 (2.59)	194 (2.60)	48 (2.57)
Total	9329 (100)	7463 (100)	1866 (100)

**Table 4.** Statistics of triplets in training and test set.

Relationship type^a^	Total, n (%)	Training set, n	Test set, n
is_examination_for	886 (18.16)	709	177
treat	839 (17.20)	671	168
is_biomarker_of	837 (17.16)	670	167
complication_of	618 (12.67)	494	124
is_located_in	579 (11.87)	463	116
is_symptom_of	449 (9.20)	359	90
is_target_of	349 (7.15%)	279	70
is_side_effect_of	303 (6.21)	242	61
increases_expression_of	19 (0.39%)	15	4
Total	4879 (100)	3903	976

a Each relationship included bidirectional relationships between head and tail entities.

### Knowledge Extraction With Different Types

LCoDR-KE achieved an overall *F*_1_ score of 81.46%. High performance was observed for the most frequent entity types, including drug (*F*_1_=95.83%) and disease (*F*_1_=90.52%), likely due to their distinct semantic boundaries and abundant contextual representations. Mid-range performance was observed for anatomy (82.38%) and test (82.11%), while categories such as gene (74.70%), complication (73.93%), and biomarker (72.03%) showed moderate degradation. The lowest entity *F*_1_ scores occurred for target (64.05%) and treatment (67.21%), not solely due to lower frequency but likely due to greater semantic ambiguity and overlapping usage with other types. Performances are detailed in Table 5.

Triplet extraction yielded an overall *F*_1_ score of 70.39% (Table 6). The model excelled on treat relations (*F*_1_=84.47%) and is_target_of (74.73%), which may benefit from clearer syntactic patterns. In contrast, performance declined for is_biomarker_of (57.69%) and increases_expression_of (50.00%), reflecting both data sparsity and higher contextual variability.

Performance differences can largely be attributed to variations in data distribution, semantic complexity, and context dependency, rather than entity or relation frequency alone. These findings suggested that targeted data augmentation, finer-grained annotation, or context-aware modeling strategies may further improve performance across challenging categories.

**Table 5.** Performance of entity extraction.

Entity type	Precision (%)	Recall (%)	*F*_1_ (%)
ALL	83.37	79.64	81.46
drug	95.44	96.23	95.83
disease	90.88	90.16	90.52
anatomy	84.23	80.60	82.38
test	82.98	81.25	82.11
side_effect	76.79	84.31	80.37
gene	64.58	88.57	74.70
complication	87.16	64.19	73.93
biomarker	75.74	68.67	72.03
symptom	66.19	76.67	71.04
treatment	70.94	63.85	67.21
target	81.67	52.69	64.05

**Table 6.** Performance of triplet extraction.

Relationship type	Precision (%)	Recall (%)	*F*_1_ (%)
ALL	73.94	67.17	70.39
treat	87.74	81.44	84.47
is_target_of	82.93	68.00	74.73
is_located_in	77.10	70.63	73.72
is_symptom_of	64.84	73.45	68.88
is_examination_for	70.47	65.63	67.96
is_side_effect_of	67.80	66.67	67.23
complication_of	80.95	55.56	65.89
is_biomarker_of	60.98	54.74	57.69
increases_expression_of	66.67	40.00	50.00

### Performance Comparisons

#### Performance Comparison With State-of-the-Art Models

To evaluate the performance, we compared our LCoDR-KE model with several state-of-the-art models, including CRF (machine learning model), BERT (general-purpose pretrained language model), BioBERT (domain-specific biomedical model), and LLMs, including DeepSeek-R1, QIANWEN, and variants with varying parameter scales. These LLMs were evaluated under a 5-shot setting, consistent with the configuration used for our LCoDR-KE model. This ensured a fair comparison under equivalent prompt-based conditions. Detailed hyperparameter settings for each model are provided in Multimedia Appendix 4.

The comparative evaluation against state‐of‐the‐art baselines (Table 7) demonstrated that LCoDR-KE delivered substantial advances in both entity and triplet extraction. Classical sequence learners, such as CRF, scored poorly (entity *F*_1_=29.18%; triplet *F*_1_=14.51%), while general‐purpose pretrained models (eg, BERT, BioBERT) achieved modest gains (entity *F*_1_≈37%; triplet *F*_1_≈30%). The Qwen2.5-7B variant improved entity extraction (*F*_1_=53.16%) but remained weak on triplets (*F*_1_=18.04%). In contrast, LCoDR-KE-0.5B reached 62.67% entity *F*_1_ and 35.05% triplet *F*_1_, and the 7B model further advanced to 81.46% and 69.04%.

Notably, at 7B parameters, LCoDR-KE approached DeepSeek-R1’s entity *F*_1_ (81.46% vs 84.64%) and marginally exceeded its triplet *F*_1_ (69.04% vs 69.02%). Although LCoDR-KE did not surpass DeepSeek-R1, it reached a similar level of effectiveness under a smaller parameter scale (7B vs 671B).

We further evaluated the OpenBioLLM-70B, which achieved an entity *F*_1_ of 59.66% (precision: 56.63%; recall: 63.04%) and a triplet *F*_1_ of 16.52% (precision: 13.92%; recall: 20.31%). In contrast, our LCoDR-KE-7B model achieved notably higher performance with 81.46% (precision: 83.37%; recall: 79.64%) in entity extraction (21.8 percentage points increase) and 69.04% (precision: 72.43%; recall: 65.96%) in triplet extraction (52.5 percentage points increase).

**Table 7.** Performance comparisons of different models.

Models	Entity extraction			Triplet extraction		
	Precision (%), mean (SD)	Recall (%), mean (SD)	*F*_1_ (%), mean (SD)	Precision (%), mean (SD)	Recall (%), mean (SD)	*F*_1_ (%), mean (SD)
Deepseek-R1	83.5 (0.082)	85.81 (0.065)	84.64^a^ (0.034)	72.36 (0.040)	65.98 (0.079)	69.02^b^ (0.096)
CRF^c^	35.65 (0.038)	24.7 (0.004)	29.18 (0.087)	18.21 (0.035)	12.06 (0.007)	14.51 (0.014)
BERT^d^	41.38 (0.085)	32.92 (0.095)	36.67 (0.084)	32.55 (0.014)	28.5 (0.004)	30.39 (0.061)
BioBERT	44.27 (0.097)	31.87 (0.019)	37.06 (0.050)	33.62 (0.004)	27.97 (0.016)	30.54 (0.004)
Qwen2.5-7B	51.75 (0.027)	54.64 (0.076)	53.16 (0.019)	16.15 (0.027)	20.44 (0.091)	18.04 (0.029)
LCoDR-KE^e^-0.5B	67.63 (0.072)	58.38 (0.046)	62.67 (0.024)	36.52 (0.083)	33.69 (0.051)	35.05 (0.018)
LCoDR-KE-1.5B	74.84 (0.055)	74.31 (0.038)	74.57 (0.085)	53.3 (0.062)	50.44 (0.007)	51.83 (0.037)
LCoDR-KE-3B	83.37 (0.006)	79.64 (0.069)	81.46^f^ (0.018)	72.43 (0.067)	65.96 (0.043)	69.04^g^ (0.094)
LCoDR-KE-7B	83.37 (0.024)	79.640.046)	81.46^f^ (0.012)	72.43 (0.023)	65.96 (0.091)	69.04^g^ (0.082)

a Best performance for entity extraction.

b Second-best performance for relationship extraction.

c CRF: conditional random fields.

d BERT: Bidirectional Encoder Representations From Transformers.

e LCoDR-KE: long chain-of-thought for drug repositioning knowledge extraction.

f Second-best performance for entity extraction.

g Best performance for relationship extraction.

#### Impact of Model Scale on Extraction Performance

**Figure 2.**
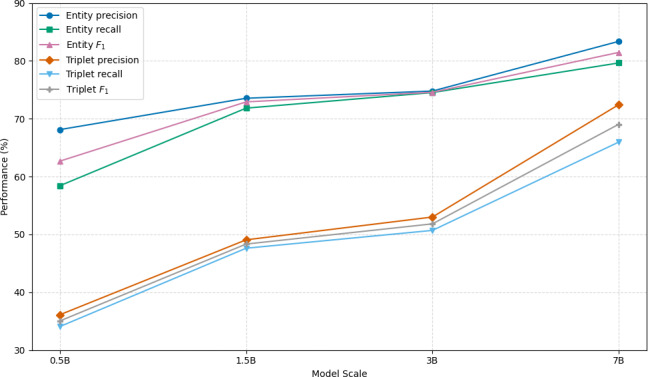
Performance of LCoDR-KE across different model scales (0.5B, 1.5B, 3B, 7B) on biomedical entity and triplet extraction tasks. Larger models yielded better precision, recall, and *F*_1_-scores. LCoDR-KE: long chain-of-thought for drug repositioning knowledge extraction.

Across the LCoDR-KE series (Figure 2), increasing model capacity from 0.5B to 1.5B, 3B, and 7B yielded consistent performance improvements, with entity *F*_1_ rising from 62.67% to 72.91%, 74.57%, and 81.46%, triplet *F*_1_ rising from 35.05% to 48.34%, 51.83%, and 69.04%, respectively. These gains suggested that larger models better capture the complex contextual dependencies required for accurate biomedical knowledge extraction.

#### Ablation Studies

To evaluate the contributions of different components within the LCoDR-KE model, we conducted ablation studies to examine the impact of strongly SFT and the LCoT model on knowledge extraction outcomes for drug repositioning. We removed the 2 components of the LCoDR-KE model to observe changes in performance.

**Figure 3.**
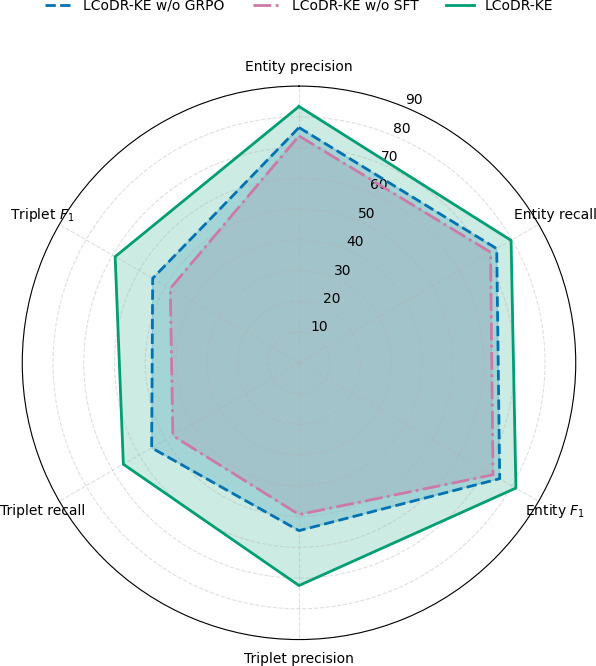
Radar chart comparing the full LCoDR-KE model with its ablation variants (without Group Relative Policy Optimization [GRPO] and without supervised fine-tuning [SFT]) across 6 evaluation metrics. Both the GRPO and SFT modules significantly enhanced performance, with SFT contributing more broadly across precision and *F*_1_-scores, especially for triplet extraction. LCoDR-KE: long chain-of-thought for drug repositioning knowledge extraction.

As presented in Table 8, the ablation studies demonstrated significant performance degradation when removing either SFT or GRPO module. Removing GRPO reduced *F*_1_-scores by 6.09% for entity extraction and 14.09% for triplet extraction, indicating that GRPO substantially enhances knowledge extraction. In comparison, removing SFT caused sharper declines in precision (8.61% for entities and 20.70% for triplets), underscoring the critical role of task‐specific supervision in adapting pretrained representations. Together, these results demonstrated that both components contribute synergistically to the model’s knowledge extraction capabilities, with GRPO enhancing reasoning and SFT improving supervised alignment. Meanwhile, the absence of SFT caused more pronounced degradation in both entity and triplet extraction, indicating its broader influence on model performance (Figure 3).

**Table 8.** Ablation study results: precision, recall, and *F*_1_-scores for different components of LCoDR-KE^a^.

Ablation models	Entity extraction			Triplet extraction		
	Precision (%)	Recall (%)	F1 (%)	Precision (%)	Recall (%)	F1 (%)
LCoDR-KE_-w/o GRPO^b^_	76.53	74.25	75.37	54.58	55.33	54.95
LCoDR-KE_-w/o SFT^c^_	73.80	71.93	72.85	49.35	47.38	48.34
LCoDR-KE	83.37	79.64	81.46	72.43	65.96	69.04

a LCoDR-KE: long chain-of-thought for drug repositioning knowledge extraction.

b GRPO: Group Relative Policy Optimization.

c SFT: supervised fine-tuning.

#### External Benchmark Evaluation

To further assess generalizability beyond our in-domain DrugReC corpus, we evaluated LCoDR-KE directly on the widely used BC5CDR benchmark, which comprises 500 PubMed abstracts manually annotated for chemical (drug) and disease entities and their binary associations. OpenBioLLM-70B achieved entity *F*_1_ of 47.39% and relation *F*_1_ of 25.52%, while DeepSeek-R1 scored 60.93% and 38.33%, respectively. Without additional fine-tuning, LCoDR-KE obtained an entity *F*_1_ of 59.17% (precision: 85.92%; recall: 45.12%) and a triplet *F*_1_ of 35.16% (precision: 33.84%; recall: 36.59%), closely matching DeepSeek-R1 and substantially outperforming OpenBioLLM-70B. Thus, our method demonstrated generalizability beyond in-domain abstracts, achieving robust performance on external biomedical corpora such as BC5CDR.

### Effect of K-Shot Examples on Model Performance

**Figure 4.**
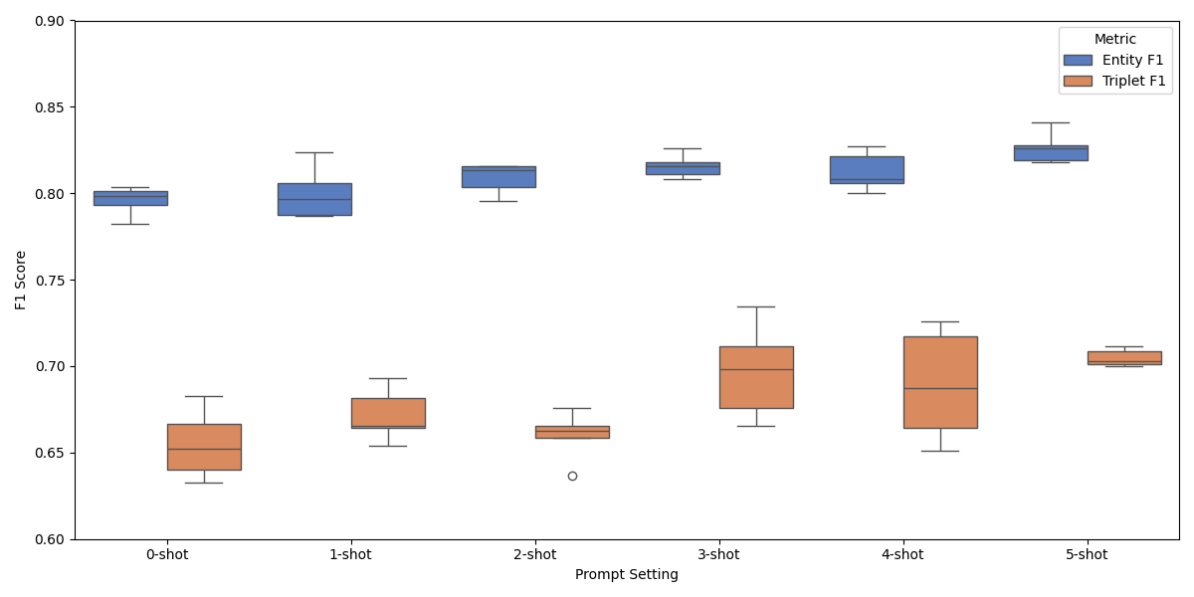
Effect of k-shot prompting on entity and triplet extraction performance. *F*_1_-scores for entity and triplet extraction across 0- to 5-shot settings. Performance improved with more shots and stabilizes between 3 shots and 5 shots.

To evaluate the impact of prompt on corpus generating, we conducted a small-scale experiment comparing 0- to 5-shot prompting settings. As shown in Figure 4, k-shot prompting consistently outperformed the 0-shot baseline in both entity and triplet extraction. Entity *F*_1_ rose from 80.01 (0 shot) to 82.63 (5 shots), while triplet *F*_1_ increased from 67.16 to 70.48. Performance steadily improved as the number of examples increased, particularly between 1 shot and 3 shots. Notably, the model achieved stable and high performance in the 3- to 5-shot range, suggesting that moderate prompt context enhances annotation quality.

### Reward Parameter Optimization

To optimize the reward function for knowledge extraction and structured reasoning, we used a progressive tuning strategy through multiple rounds of controlled experiments. The reward formulation includes a hierarchical accuracy component where the coefficients for entity recognition (α), triplet extraction (β), and rare entity/relation enhancement (γ) satisfy the constraint α + β + *γ* = 1. Given the high computational cost of jointly searching in a 3D space, we adopted a 2-stage approach. We first optimized α and β under the constraint α + *β* = 1 to balance entity- and relation-level performance. Once optimal base weights were identified, we introduced γ by gradually adjusting the proportion of reward allocated to rare cases. Subsequently, we adjusted the weights associated with structured reasoning, namely tag integrity (δ) and reasoning length (ε), to ensure syntactic validity and controlled verbosity in the model’s output. Finally, we calibrated the overall balance between accuracy-related rewards and format-related rewards by modifying the global weight of accuracy reward and format reward parameters.

In addition, we introduced 2 top-level coefficients (ie, accuracy reward weight and format reward weight) to control the relative contribution of accuracy-based and format-based rewards in the overall objective. These weights were jointly tuned to ensure a trade-off between extraction performance and output structure quality.

Throughout the process, evaluation was based on both entity and relation *F*_1_-scores, as well as the structural completeness of generated outputs (Multimedia Appendix 5). The final parameter configuration, with *α*=.45, *β*=.45, *γ*=0.1, *δ*=0.7, *ε*=0.3, accuracy reward=0.5, and format reward=0.5, demonstrated optimal performance in terms of extraction accuracy, robustness, and output interpretability and was therefore adopted in subsequent experiments.

### Interpretable CoT Reasoning Patterns

To evaluate whether the model engaged in structured reasoning, we examined its CoT outputs during knowledge extraction. We gave an example (Table 9) to demonstrate that the model performed structured, schema-aware reasoning beyond pattern matching. It explicitly validated entity types, rejected schema-incompatible relations, and enforced output constraints, ultimately generating a compliant JSON structure. The complete example of CoT is summarized in Multimedia Appendix 6.

**Table 9.** Structured chain-of-thought reasoning example from LCoDR-KE^a^.

Reasoning step	Model output (excerpt)
Task framing	“Alright, I’m approaching this task as a biomedical knowledge extraction assistant. I need to systematically identify entities and relationships from the predefined types.”
Entity identification	“cetuximab’ clearly fits the Drug definition as a therapeutic agent; ‘colorectal cancer’ → Disease; ‘infusion reactions’ → Side Effect; ‘ER visit’ → Treatment...”
Entity filtering	“‘medical intervention’ is too vague–not entity material;...”
Relation reasoning	“‘cetuximab’ causes ‘infusion reactions’ → is_side_effect_of; ‘cetuximab’ treats ‘colorectal cancer’ → treat...”
Schema constraints	“Rejected is_examination_for between ER and IRs – not diagnostic; did not create invented relations like ‘discontinued_due_to’...”
Formatting awareness	“Ensured JSON format with two root keys (Entities and Relationships); original casing preserved; no explanatory text embedded...”

a LCoDR-KE: long chain-of-thought for drug repositioning knowledge extraction.

### Step of Training Iterations

**Figure 5.**
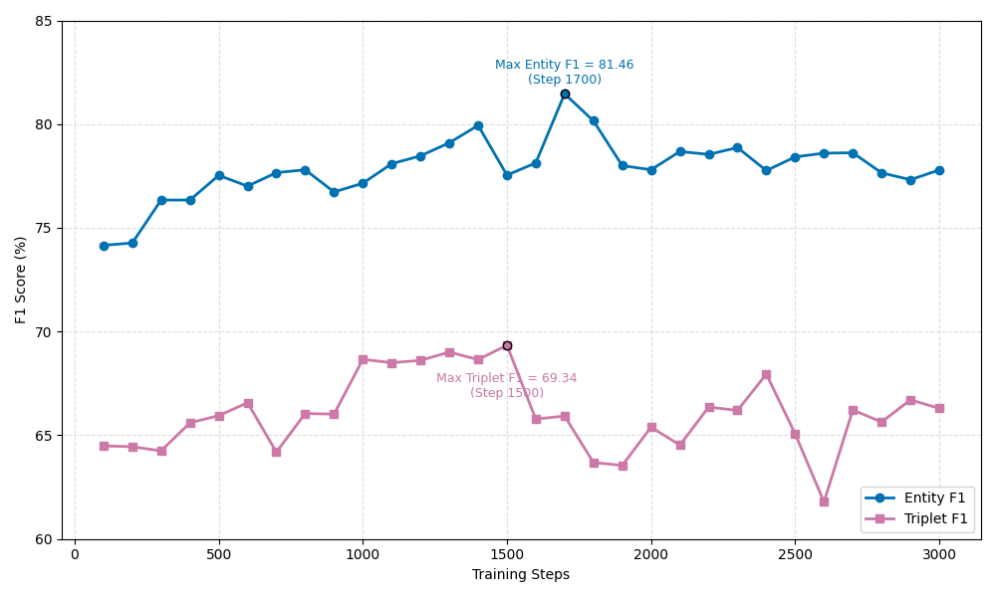
Entity and triplet F1 performance across 30 training iterations (100‐3000 steps). Entity *F*_1_ steadily improved and peaked at 81.46% (step 1700), maintaining stability thereafter. Triplet *F*_1_ reached a maximum of 69.34% (step 1500) and remained consistently above 65%, indicating robust and stable model convergence for both extraction tasks.

We also monitored the performance across 30 training iterations (from 100 to 3000 steps) using *F*_1_ metrics (Figure 5) and showed a steady and stable improvement in Entity *F*_1_, which reached a peak of 81.46% at the level of 1700. Notably, Entity *F*_1_ remained consistently high beyond 2000 iterations without significant decline, indicating robust and stable convergence in the entity recognition task. Triplet *F*_1_ reached its highest value of 69.34% at step 1500 and exhibited a slight decrease in subsequent iterations; however, it consistently stayed above 65%, suggesting that the model maintained a strong baseline for triplet extraction. This relatively stable performance postpeak indicated the potential for further optimization with continued training or minor adjustments. Overall, these results demonstrated the effectiveness and stability of the training approach, particularly for entity recognition while also highlighting a promising and resilient trajectory for triplet extraction performance. Detailed performances across training iterations are provided in Multimedia Appendix 7.

### Error Analysis

We conducted an error analysis by manually reviewing 100 randomly selected sentences from the model outputs to identify common types of mistakes and areas for improvement.

#### Error Examples

The errors were classified into 4 main types of entity extraction (ie, incorrect extraction, incorrect type, boundary errors, and missing entities) and 2 main types of triplet extraction (incorrect entity type and cross-sentence errors), with examples summarized in Table 10.

**Table 10.** Main error types in LCoDR-KE^a^.

Error types	Definition	Sentences	Truth extraction	LCoDR-KE extraction
Entity extraction				
Incorrect extraction	Nonentity terms were incorrectly identified as entities	Available therapies are effective, associated with severe side effects.	NULL	[severe side effects, side effect]
Incorrect type	Entities were correctly identified but assigned the wrong entity type	In nearly half of cancer cells, there is an overexpression of MDM2 and MDMX, which inhibit p53 activity.	[p53, gene]	[p53, target]
Boundary errors	Which involved incorrect determination of the start and end positions of entities	The expression of BRCA1 and BRCA2 mutations was analyzed in the cohort.	[BRCA2, gene]	[BRCA2 mutations, gene]
Missing entities	Entities present in the text were not identified by the model	Other lung findings were ... or extensive hemorrhage.	[extensive hemorrhage, complication]	NULL
Triplet extraction				
Incorrect entity type	Relationships were correctly identified but assigned the wrong entity type	Docetaxel and gemcitabine might result in fewer adverse events.	NULL	[adverse events, side effect, is side effect of, Docetaxel, drug]
Cross- sentence error	Unable to recognize logical relationships across sentences	62% of subjects exhibited recurrent gastrointestinal distress (eg, nausea). Subsequent serological testing confirmed elevated anti-tTG antibodies, a hallmark of celiac disease.	[nausea, symptom, is symptom of, celiac disease, disease]	NULL

a LCoDR-KE: long chain-of-thought for drug repositioning knowledge extraction.

#### Error Statistics

We further quantified the frequency of each error type to assess their impact and guide future improvement strategies.

For entity extraction:

1. Incorrect extraction (n=10, 38.46%): This was the most common error, typically caused by semantically ambiguous phrases. For example, generic terms such as “severe side effects” were incorrectly extracted due to superficial similarity to defined entity types.
2. Incorrect type (n=6, 23.08%): These errors occurred when the model misclassified closely related concepts, such as mislabeling “p53” as a target instead of a gene.
3. Boundary errors (n=4, 15.38%): Often caused by inclusion of modifiers like “mutations” in entity spans, resulting in misaligned entity boundaries.
4. Missing entities (n=6, 23.08%): Frequently occurred when entities were embedded in complex phrases or lacked explicit semantic cues, leading to omission.

For triplet extraction:

1. Incorrect entity type (n=5, 38.46%): The relationship was correctly identified, but the entity types were misclassified (eg, “adverse events” mislabeled as side effect instead of a nonentity).
2. Cross-sentence error (n=8, 61.54%): The model failed to capture logical relationships spanning multiple sentences, especially when the subject and object were not co-located in the same sentence.

**Figure 6.**
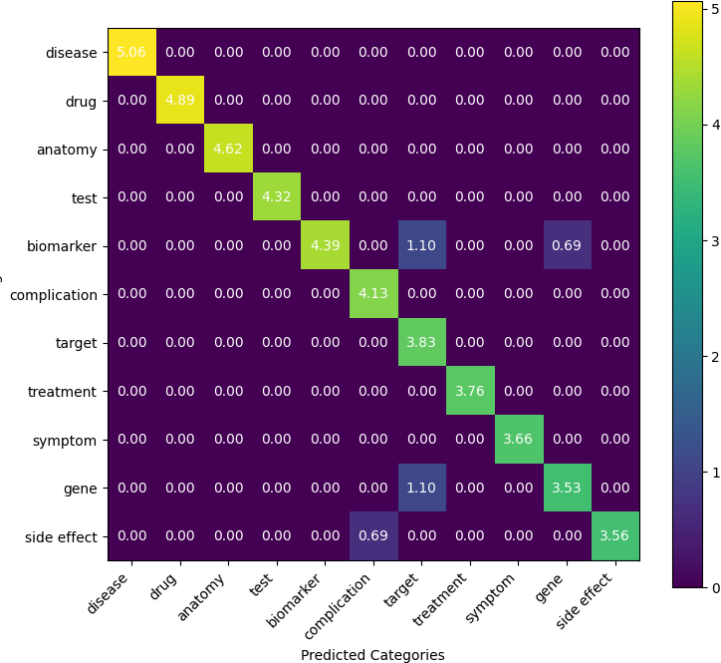
Confusion matrix of entity type classification results (log-normalized). This log-scaled confusion matrix illustrated the model's entity classification performance across 11 biomedical entity types. Diagonal dominance indicated high overall accuracy, while off-diagonal cells highlight common misclassifications. Notably, errors were concentrated among semantically similar categories, such as biomarker, gene, and target.

To further investigate the model’s behavior on entity classification, we constructed a confusion matrix (Multimedia Appendix 8) based on entity predictions. The results (Figure 6) showed strong diagonal dominance, indicating high accuracy across most entity types. However, misclassifications persist among semantically similar categories. Specifically, the biomarker was confused with the target (2 cases) and gene (1 case), while gene was misclassified as the target twice. In addition, 1 side effect instance was incorrectly labeled as a complication. These errors reflected the model’s difficulty in distinguishing biologically related concepts with overlapping semantics. In contrast, core categories, such as drug and disease, were classified with perfect accuracy.

#### Comparisons Between LCoDR-KE and DeepSeek-R1

Meanwhile, LCoDR-KE demonstrated comparable performance with DeepSeek-R1. Specifically, LCoDR-KE surpassed DeepSeek-R1 in the categories of incorrect type (6 vs 8), missing entity (6 vs 7), and incorrect entity type (5 vs 6). This advantage can be attributed to our meticulous definition of entities and relations for the drug repurposing task, as well as the provision of accurate examples during model training. Conversely, LCoDRKE performed slightly worse in the Incorrect Extraction (10 vs 8) and CrossSentence Error (8 vs 7) categories, indicating that its complex contextual understanding and reasoning capabilities warrant further improvement.

## Discussion

### Principal Findings

In this study, we introduced LCoDR-KE, a novel framework for drug repositioning knowledge extraction that significantly enhanced the adaptive learning capabilities of LLMs.

The effectiveness of our approach stemmed from 2 key innovations.

- First, we developed a refined semantic representation framework and used it to curate a high-quality training corpus, DrugReC, comprising 1000 expertly annotated abstracts. These annotations covered diverse drug repositioning entities and captured complex semantic relationships, providing a robust foundation for the model to learn accurate semantic understanding and contextual reasoning.
- Second, we proposed a long reasoning chain strategy: an iterative, multistep inference process reinforced through reward mechanisms and RL. By delivering explicit feedback at each reasoning step, this strategy enabled the model to progressively optimize its decision-making process, resulting in more accurate and coherent inference.

Collectively, these innovations contributed to the improved performance of LCoDR-KE in extracting drug repositioning knowledge.

Compared to pretraining approaches specifically designed for biomedical tasks, LCoDR-KE offered several advantages. Traditional methods typically required extensive domain adaptation, large annotated corpora, or multiple rounds of fine-tuning to achieve competitive performance [55]. In contrast, LCoDR-KE achieved performance optimization with a relatively small training dataset—comprising only 1000 literature abstracts—thereby significantly reducing the human and time costs associated with domain-specific annotation. Furthermore, LCoT decomposed semantic understanding into explicit and traceable reasoning steps. This approach mitigated hallucinations and enhanced model transparency, addressing 2 major challenges in the application of LLMs. Our ablation study further substantiated these findings.

Moreover, performance comparisons demonstrated that LCoDR-KE based on the Qwen-2.5-7B model matched the performance of the 671B -parameter DeepSeek-R1 in drug repurposing knowledge extraction. Specifically, *F*_1_-scores achieved 81.46% versus 84.64% for entity extraction and 69.04% versus 69.02% for triplet extraction. This advance significantly narrowed the gap between large-scale models and resource-constrained environments, making it a practical solution for research institutes and hospital information centers with modest computing capabilities.

In addition to performance gains, LCoDR-KE enhanced model interpretability through CoT prompting. By generating intermediate reasoning steps, such as entity justification, schema validation, and relation filtering, the model made its decision process more transparent and traceable. This structured reasoning not only aided error diagnosis but also aligned model behavior with domain knowledge constraints, offering a more controllable and explainable framework for biomedical knowledge extraction.

Our expanded error analysis provided a more nuanced understanding of model behavior in biomedical knowledge extraction. By categorizing common error types, quantifying their frequency, and visualizing entity-level confusion patterns, we identified key challenges such as semantic ambiguity, long-range dependencies, and data sparsity. These insights not only explained performance bottlenecks but also informed future improvements, such as targeted data augmentation and schema-aware prompt refinement to reduce ambiguity and improve generalization.

LCoDR-KE demonstrated strong performance in low-resource settings through its schema design, cold-start SFT, and RL with a reward mechanism. It provides an efficient and feasible solution for biomedical knowledge extraction under resource-constrained conditions. In clinical research settings, LCoDR-KE could be deployed to mine structured knowledge from trial protocols, real-world evidence, or adverse event reports, assisting clinicians in therapeutic decision-making and safety monitoring. In early-stage drug development, it can support target identification and biomarker discovery by extracting mechanistic insights from scientific literature. Its lightweight design makes it particularly well suited for integration into domain-specific platforms where computational resources and labeled data are limited. Moreover, its task-specific schema could be readily tailored by redefining entities and relations, thereby accommodating new domains, languages, and even alternative LLM backbones. Its flexibility allowed LCoDR-KE to extend beyond drug repositioning to a broad range of biomedical knowledge extraction tasks.

### Limitations

Despite the promising results, our study has some limitations. First, we limited the task to drug repositioning in this study. In the future, we will include other biomedical knowledge extraction tasks, such as gene–disease association identification and drug–drug interaction detection, thereby expanding its application value in diverse scenarios. Second, the model’s balance between reasoning depth and computational efficiency remains suboptimal, particularly when processing intricate biomedical contexts requiring multistep logical inferences. Future optimizations will focus on lightweight architectural improvements.

### Conclusions

This study introduced LCoDR-KE, a framework that enhanced LLMs’ domain-specific adaptability for drug repositioning by integrating long-chain reasoning and RL. We constructed a publicly available DrugReC and a lightweight knowledge extraction model, offering critical resources for accelerating drug discovery and knowledge inference. The framework balances accuracy, interpretability, and computational efficiency, validated through stable training convergence and competitive performance against state-of-the-art models. Overall, LCoDR-KE not only advances drug repositioning research but also provides a scalable, transferable methodology applicable to diverse biomedical knowledge extraction tasks, knowledge reasoning, and even decision support, bridging gaps in low-resource, specialized domains.

## Supplementary material

10.2196/77837 Multimedia Appendix 1 List of drug repositioning journal and PRISMA (Preferred Reporting Items for Systematic reviews and Meta-Analyses) guidelines.

10.2196/77837 Multimedia Appendix 2 Chain-of-thought prompt.

10.2196/77837 Multimedia Appendix 3 Drug repositioning schema.

10.2196/77837 Multimedia Appendix 4 Hyperparameters of fine-tuned models.

10.2196/77837 Multimedia Appendix 5 Reward weight.

10.2196/77837 Multimedia Appendix 6 Reasoner.

10.2196/77837 Multimedia Appendix 7 Performance across training iterations.

10.2196/77837 Multimedia Appendix 8 Confusion matrix.

## Abbreviations

CoT: chain-of-thought
CRF: conditional random fields
DrugReC: drug repositioning corpus
GRPO: Group Relative Policy Optimization
LCoDR-KE: drug repositioning knowledge extraction model based on long chain-of-thought
LCoT: long chain-of-thought
LLM: large language model
MeSH: Medical Subject Headings
PRISMA: Preferred Reporting Items for Systematic Reviews and Meta-Analyses
RL: reinforcement learning
SFT: supervised fine-tuning
