# Supplementary material for: Large Language Model–Enhanced Drug Repositioning Knowledge Extraction via Long Chain-of-Thought: Development and Evaluation Study
Source: JMIR Med Inform. 2025 Oct 7;13:e77837. doi: 10.2196/77837 (PMC12503436; doi:10.2196/77837)

| No. | Journal Name | IF Score | Rank |
| --- | --- | --- | --- |
| 1 | Nature Reviews Drug Discovery | 122.7 | Q1 |
| 2 | Pharmacological Reviews‌ | 19.3 | Q1 |
| 3 | Drug Resistance Updates‌ | 15.8 | Q1 |
| 4 | Drug Resistance Update | 15.8 | Q1 |
| 5 | Advanced Drug Delivery Reviews‌ | 15.2 | Q1 |
| 6 | Annual Review of Pharmacology and Toxicology‌ | 11.2 | Q1 |
| 7 | Journal of Controlled Release | 10.5 | Q1 |
| 8 | Journal for ImmunoTherapy of Cancer‌ | 10.3 | Q1 |
| 9 | Pharmacological Research‌ | 9.1 | Q1 |
| 10 | BIOMEDICINE & PHARMACOTHERAPY‌ | 6.9 | Q1 |
| 11 | Briefings in Bioinformatics | 6.8 | Q1 |
| 12 | British Journal of Pharmacology‌ | 6.8 | Q1 |
| 13 | Drug Discovery Today | 6.5 | Q1 |
| 14 | Journal of Translational Medicine | 6.1 | Q1 |
| 15 | Drug Delivery and Translational Research | 5.7 | Q1 |
| 16 | Biochemical Pharmacology | 5.3 | Q1 |
| 17 | Journal of Ethnopharmacology | 4.8 | Q1 |
| 18 | European Journal of Pharmacology | 4.2 | Q1 |


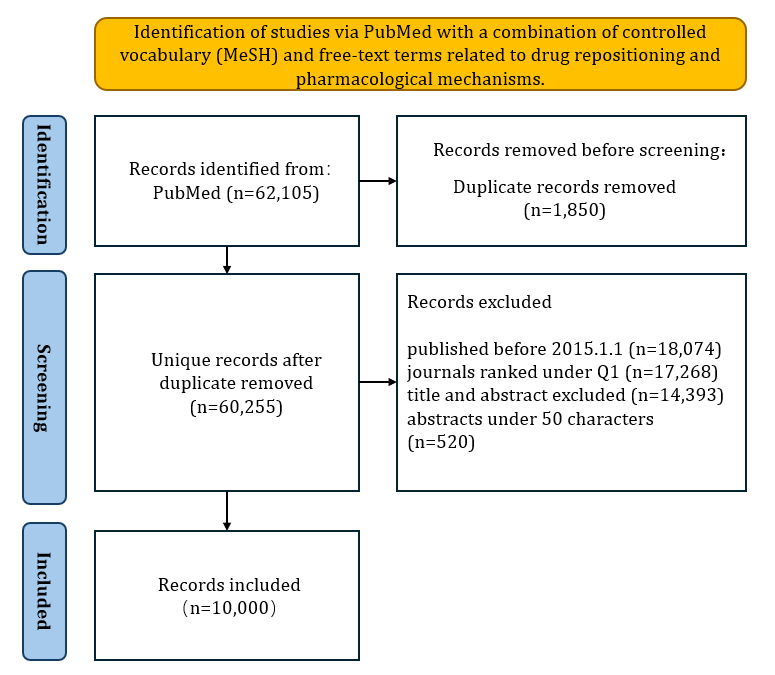

Supplement: Multimedia Appendix 1 [file medinform-v13-e77837-s001.docx]
