# Supplementary material for: Large Language Model–Enhanced Drug Repositioning Knowledge Extraction via Long Chain-of-Thought: Development and Evaluation Study
Source: JMIR Med Inform. 2025 Oct 7;13:e77837. doi: 10.2196/77837 (PMC12503436; doi:10.2196/77837)
Supplement: Multimedia Appendix 4 [file medinform-v13-e77837-s004.docx]

| Hyperparameter | Model size | Max_seq_len | Learning_rate | Batch_size | Epoch | Optimizer | Finetunig_type |
| --- | --- | --- | --- | --- | --- | --- | --- |
| CRF | 3M | 512 | 0.00005 | 50 | 50 | Adam | full |
| BERT | 110M | 512 | 0.00005 | 50 | 50 | Adam | full |
| BioBERT | 110M | 2048 | 0.00005 | 8 | 15 | Adam | full |
| Qwen2.5-7B | 7B | 16384 | 0.00005 | 8 | 15 | Adam | full |
| LCoDR-KE-0.5B | 0.5B | 16384 | 0.000001 | 8 | 15 | Adam | full |
| LCoDR-KE-1.5B | 1.5B | 16384 | 0.000001 | 8 | 15 | Adam | full |
| LCoDR-KE-3B | 3B | 16384 | 0.000001 | 8 | 15 | Adam | full |
| LCoDR-KE-7B | 7B | 16384 | 0.000001 | 8 | 15 | Adam | full |
